# Development of a novel BRCAness score that predicts response to PARP inhibitors

**DOI:** 10.1186/s40364-022-00427-8

**Published:** 2022-11-12

**Authors:** Masanori Oshi, Shipra Gandhi, Rongrong Wu, Mariko Asaoka, Li Yan, Akimitsu Yamada, Shinya Yamamoto, Kazutaka Narui, Takashi Chishima, Takashi Ishikawa, Itaru Endo, Kazuaki Takabe

**Affiliations:** 1 Department of Surgical Oncology, Roswell Park Comprehensive Cancer Center, Buffalo, NY 14263 USA; 2 Department of Gastroenterological Surgery, Yokohama City University Graduate School of Medicine, Yokohama, 236-0004 Japan; 3 Department of Medical Oncology, Roswell Park Comprehensive Cancer Center, Buffalo, NY 14263 USA; 4 Department of Breast Surgery and Oncology, Tokyo Medical University, Tokyo, 160-8402 Japan; 5 Department of Biostatistics & Bioinformatics, Roswell Park Comprehensive Cancer Center, Buffalo, NY 14263 USA; 6 Department of Breast and Thyroid Surgery, Yokohama City University Medical Center, Yokohama, Kanagawa Japan; 7 Division of Digestive and General Surgery, Niigata University Graduate School of Medical and Dental Sciences, Niigata, 951-8520 Japan; 8 Department of Breast Surgery, Fukushima Medical University School of Medicine, Fukushima, 960-1295 Japan; 9 Department of Surgery, Jacobs School of Medicine and Biomedical Sciences, State University of New York, Buffalo, NY 14263 USA; 10 Department of Breast Surgery, Roswell Park Comprehensive Cancer Center, Elm & Carlton Streets, Buffalo, NY 14263 USA

**Keywords:** Breast cancer, BRCAness, Gene expression, Signaling, Biomarker

## Abstract

**Background:**

BRCAness is a characteristic feature of homologous recombination deficiency (HRD) mimicking BRCA gene mutation in breast cancer. We hypothesized that a measure to quantify BRCAness that causes synthetic lethality in BRCA mutated tumors will identify responders to PARP inhibitors.

**Methods:**

A total of 6753 breast cancer patients from 3 large independent cohorts were analyzed. A score was generated by transcriptomic profiling using gene set variation analysis algorithm on 34 BRCA1-mutation related genes selected by high AUC levels in ROC curve between BRCA1 mutation and wildtype breast cancer.

**Results:**

The score was significantly associated with BRCA1 mutation, high mutation load and intratumoral heterogeneity as expected, as well as with high HRD, DNA repair and MKi67 expression regardless of BRCA mutations. High BRCAness tumors enriched not only DNA repair, but also all five Hallmark cell proliferation-related gene sets. High BRCAness tumors were significantly associated with higher cytolytic activity and with higher anti-cancerous immune cell infiltration. Not only did the breast cancer cell lines with BRCA-mutation show high score, but even the other cells in human breast cancer tumor microenvironment were contributing to the score. The BRCAness score was the highest in triple-negative breast cancer consistently in all 3 cohorts. BRCAness was associated with response to chemotherapy and correlated strongly with response to PARP inhibitor in both triple-negative and ER-positive/HER2-negative breast cancer.

**Conclusions:**

We established a novel BRCAness score using BRCA-mutation-related gene expressions and found that it associates with DNA repair and predicts response to PARP inhibitors regardless of BRCA mutation.

**Supplementary Information:**

The online version contains supplementary material available at 10.1186/s40364-022-00427-8.

## Background

BRCAness is a phenotype mimicking mutations of germline *BRCA1* and/or *BRCA2* DNA repair gene [1] that are involved in all phases of the cell cycle [2], which results in homologous recombination deficiency (HRD). For instance, somatic mutations of homologous recombination repair (HRR) genes such as ATM, ATR, PALB2 and RAD51 cause BRCAness [3]. BRCAness was reported to predict response to anticancer agents [4]. Since poly ADP-ribose polymerase (PARP) is also essential in DNA repair, PARP inhibitors (PARPis) cause synthetic lethality in tumors with *BRCA1* and/or *BRCA2* germline mutations and are used for ovarian, prostate, pancreatic, and breast cancer. There is a growing interest whether PARPis could be used for high BRCAness patients regardless of BRCA gene mutations.

To this end, quantification of BRCAness in breast cancer is expected to guide the use of PARPis. Although several parameters have been reported to estimate BRCAness, currently the indication of PARPis in clinical practice is still limited to patients with BRCA germline mutation status. Given the “central dogma” of molecular biology that cellular phenotype is determined from DNA to protein through mRNA expression, we hypothesized that a score generated by a gene expression profile that represents *BRCA* gene mutation will allow us to quantify BRCAness and predict response to agents including PARPis.

Here, we aimed to establish a novel BRCAness score with BRCAness-related genes by GSVA algorithm and study the clinical relevance of BRCAness in breast cancer. We hypothesized that the BRCAness score, which strongly reflects BRCAness, is associated with tumor immune microenvironment, tumor aggressiveness, clinical outcomes, and prediction of response to PARPis.

## Materials and methods

### Data acquisition of breast cancer

Clinical information and gene expression data were obtained from 1903 breast cancer patients in the Molecular Taxonomy of Breast Cancer International Consortium (METABRIC) cohort through cBioportal [5, 6]. Clinical information and gene expression data of 3273 breast cancer patients in the GSE96058 cohort was obtained from the Swedish Breast Cancer Analysis Network (SCAN-B) [7]. Clinical and transcriptomic data was also obtained on 1069 female breast cancer patients from The Cancer Genome Atlas (TCGA) cohort [8]. The Biospecimen Core Resource collected and processed the frozen samples from treatment naive breast cancers for the TCGA project [9, 10]. Genome Sequencing Centers and Genome Characterization Centers conducted the RNA-sequencing. The TCGA data was made publicly available by the TCGA Research network. Gene expression data were obtained in RSEM format and converted to Transcripts Per Million (TPM) by a given gene’s estimated fraction of transcripts and multiplying by 10^6^. The GSE75688 cohort has single-cell RNA-sequencing data of tumor, stromal, immune, and myeloid cells in breast cancer [11], which was obtained from Gene Expression Omnibus (GEO). Because all the data obtained from METABRIC, GSE96058 and TCGA are deidentified and displayed in public domain, the Institutional Review Board of Roswell Park approval was waived.

### DNA repair signaling score

To estimate the activity level of DNA repair, we used the Hallmark DNA repair gene set, which have 150 DNA repair-related genes (supplementary table S1), in the Molecular Signatures Database (MSigDB) calculated using by Gene Set Variation Analysis (GSVA) algorithm [12, 13], as we previously reported [14].

### Other scores

The cytolytic activity score (CYT) was calculated using expression levels of perforin (*PRF1*) and granzyme A (*GZMA*) [15]. CYT was used to quantify immune cytolytic activity in tumor microenvironment (TME) rich in T cells as we have shown in another study [16]. The fraction of 64 infiltrating stromal and immune cells in each tumor were estimated by gene expression profiles using xCell algorithm [17], as we previously reported [18–20]. The calculations reported by Thorsson et al. [21] in the TCGA cohort were used to analyze the mutation-related scores; fraction altered, homologous recombination defects (HRD), silent and non-silent mutation rate, single nucleotide variation (SNV), indel neoantigens, and intratumor heterogeneity.

### Gene set enrichment analysis

Gene Set Enrichment Analysis (GSEA) Java software (vers. 4.1) was used to conduct GSEA [22] using Hallmark collection of MSigDB gene sets [13] to explore the cancer biology that enrich to either high or low BRCAness score patients. False discovery rate (FDR) < 25% was used to deem statistical significance, as recommended by GSEA.

### Statistical analyses

All analyses and data plots were conducted using R software (vers. 4.1.0) and Microsoft Excel (vers. 16). The top tertile was defined as high BRCAness group in each cohort (Fig. S1). The Fisher exact test, the Mann-Whitney U test, or the Kruskal-Wallis test were performed for group comparison analyses. Survival analysis between two groups was shown using the Kaplan-Meier plot with the log-rank test. Values of *p* < 0.05 indicate a statistically significant difference.

## Results

### The novel BRCAness score was established using 34 genes with high area under the curve value for BRCA1-mutation in both TCGA and METABRIC cohorts

**Fig. 1:**
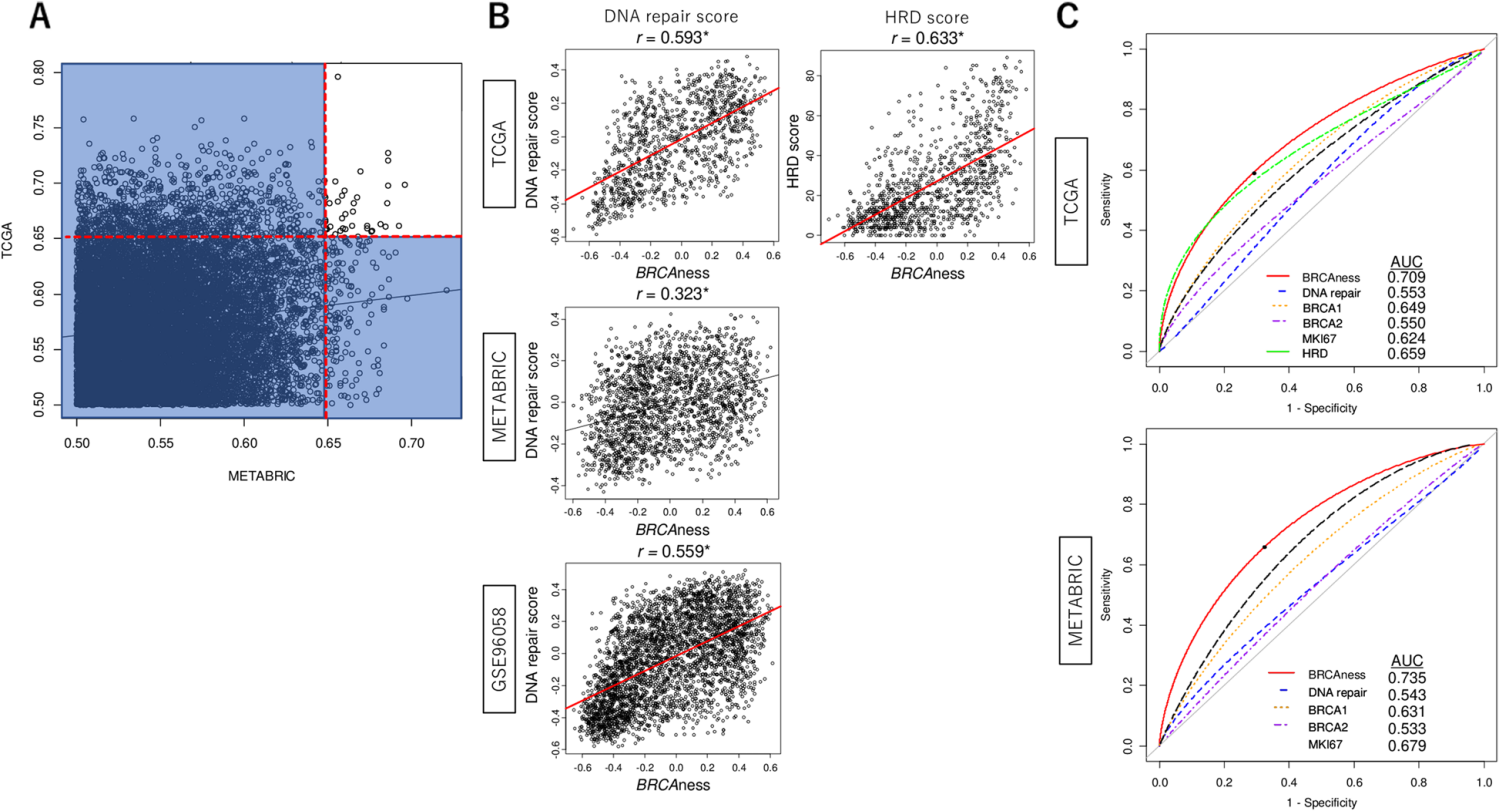
Development of the BRCAness score that correlates with DNA repair and HRD, which has the highest AUC for BRCA1 mutation, in breast cancer. **A** Correlation plots of the AUC level of the BRCAness score with BRCA1 mutation in the METABRIC and TCGA cohorts by Spearman’s rank correlation test. Blue shaded areas represent AUC lower than 0.65 in either TCGA or METABRIC. **B** Scatter plots of the score level between the BRCAness score and DNA-repair score in the TCGA, METABRIC, and GSE96058 cohorts, and HRD score in the TCGA cohort. Spearman’s rank correlation was used for the analysis. **C** ROC curve of the BRCAness score, DNA repair score, *BRCA1* and *BRCA2* gene expression, *MKI67* expression, and HRD score, with the AUC in the TCGA and METABRIC cohorts. AUC, area under the curve; HRD, homologous recombinant deficient; ROC, receiver operating characteristic

We analyzed the mRNA expression of all genes for patients with *BRCA1*-mutation and wildtype by receiver operating characteristic-area under the curve (ROC-AUC). We found that the expressions of 34 genes were significantly associated with *BRCA1*-mutation consistently with the AUC level in both cohorts (Fig. 1A and Table S2; AUC > 0.650). BRCAness score was generated with these 34 genes using GSVA algorithm, similar to our previous works [14, 23–25]. In order to assess the performance of this BRCAness score, its correlation with DNA repair that is known to be associated with BRCAness, was evaluated. The DNA repair score was calculated by GSVA algorithm using the “Hallmark DNA repair” gene set of the molecular signatures database (MSigDB) as we previously reported [14]. The BRCAness score correlated highly with DNA repair score in TCGA and GSE96058, but not in METABRIC (Fig. 1B, Spearman rank correlation (*r*) = 0.593, 0.559, and 0.323, respectively). The correlation between the BRCAness score and HRD score, which were pre-calculated for the patients in the TCGA by Thorsson et al. [21], was also assessed. BRCAness and HRD scores were strongly correlated in TCGA (Fig. 1B, *r* = 0.633). Next, to assess the predictive performance of BRCAness score with *BRCA1* mutation, ROC-AUC analysis was performed. We found that AUC of BRCAness score was 0.709 and 0.735 in TCGA and METABRIC, respectively (Fig. 1C). These levels were highest compared to other AUC of BRCAness-related factors, including DNA repair score, *BRCA1* and *BRCA2* gene expression, *MKI67*, and HRD score in the TCGA cohort. These results were validated in the METABRIC cohort. The difference in the relationship of BRCAness score with DNA repair (Fig. 1B) vs. ROS (Fig. 1C) in METABRIC may be explained by the fact that BRCAness is not only a mere reflection of DNA repair.

### High BRCAness was significantly associated with BRCA1 mutation and high mutation load, as well as high level of DNA repair and MKi67 expression regardless of BRCA1 mutation

**Fig. 2:**
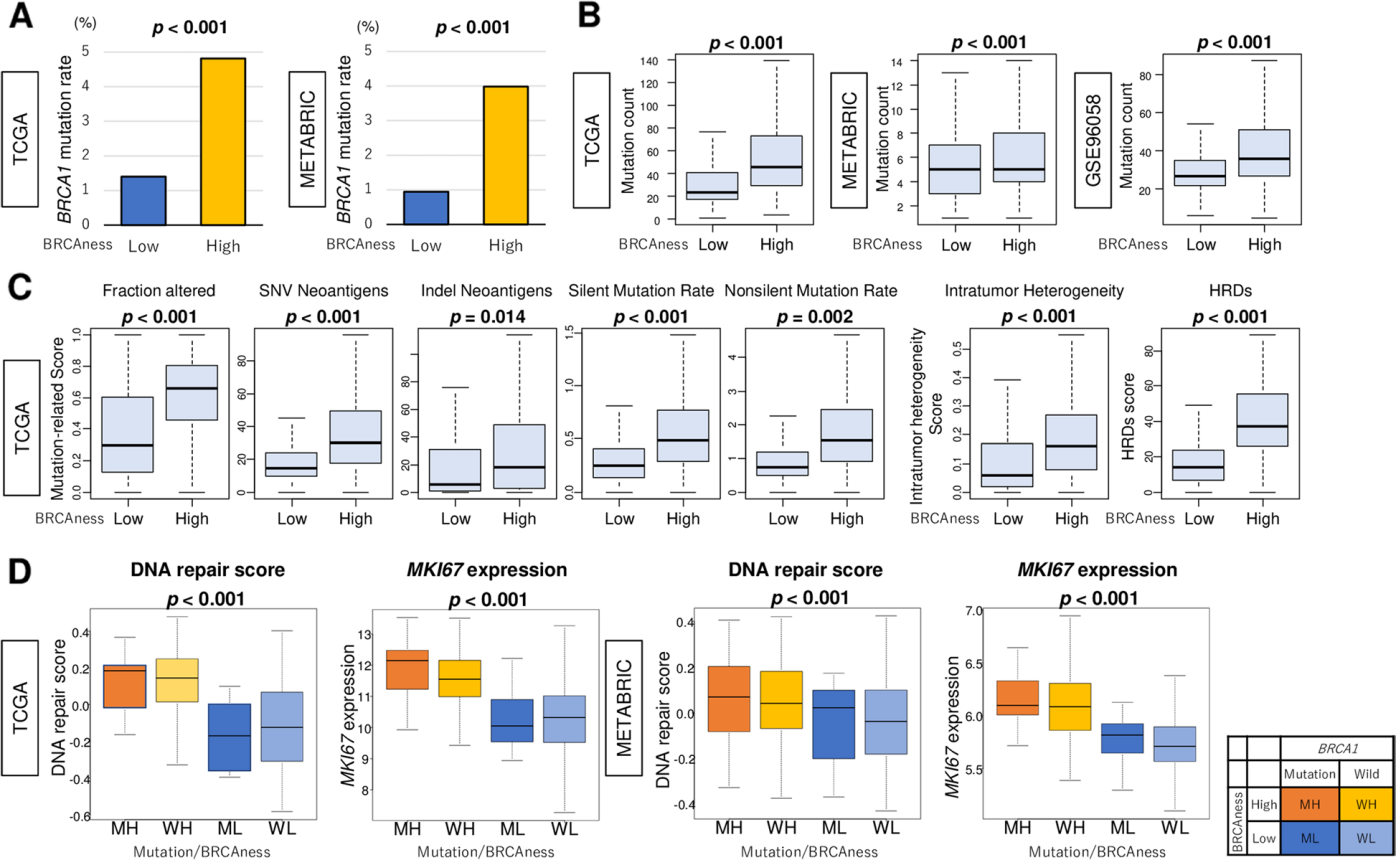
Association of BRCAness score with mutation load, intratumor heterogeneity, and BRCA1 mutation with DNA repair and MKI67 expression in breast cancer. **A** Bar plots of BRCA1 mutation rates by low or high BRCAness in the METABRIC and TCGA cohorts. Fisher’s exact test was used to calculate *p*-values. **B** Boxplots of mutation counts (per mega base) by low or high BRCAness groups in the GSE96058, METABRIC, and TCGA cohorts. **C** Boxplots of mutation-related scores; fraction altered, silent & non-silent mutation rate, SNV & indel neoantigens, intratumor heterogeneity, and HRD, by low or high BRCAness groups. **D** Boxplots of the DNA repair score and MKI67 gene expression by four groups (MH; BRCA1 mutation with high BRCAness, WH; BRCA1 wildtype with high BRCAness, ML; BRCA1 mutation with low BRCAness, and WL; BRCA1 wildtype with low BRCAness) in the TCGA (*n* = 17/336/10/706) and METABRIC (*n* = 25/603/12/1264) cohorts. The top tertile was used as a cutoff to divide high- from low- BRCAness groups. *T*he Mann-Whitney U test and Kruskal-Wallis test were used to calculate *p*-values. SNV, single nucleotide variation

High BRCAness score was significantly associated with high *BRCA1* mutation rate in both the METABRIC and TCGA cohorts (Fig. 2A; both *p* < 0.001), however, High BRCAness score was associated with a trend towards higher BRCA2 mutation ratewithout reaching statistical significance in either of the cohorts (Fig. S2). We found that high BRCAness was associated significantly with high mutation counts consistently in three cohorts (Fig. 2B; *p* < 0.001). Furthermore, we investigated the association of the BRCAness score with mutation-related scores: fraction altered, silent and non-silent mutation rate, single nucleotide variation and indel neoantigens, as well as intratumor heterogeneity, and HRD. We found that breast cancer with high BRCAness was associated significantly with high mutation rates, intratumor heterogeneity and HRD score (Fig. 2C).

Next, we compared the distribution of high and low BRCAness score with DNA repair score and *MKi67* expression with or without BRCA1-mutation in breast cancer. The top tertile was defined as high BRCAness groups in each cohort. We found that both DNA repair and *MKi67* expression were higher in high vs. low BRCAness group in *BRCA1* mutation breast cancer in the TCGA cohort (Fig. 2D). There was no difference in either the DNA repair score or *MKI67* expression by *BRCA1* mutation and wildtype regardless of the level of BRCAness score. These results were validated by the METABRIC cohort and suggest that BRCAness score associates better with DNA repair and *MKi67* expression compared with *BRCA1* mutation status.

### DNA repair and cell proliferation-related gene sets were significantly enriched in high BRCAness breast cancer

**Fig. 3:**
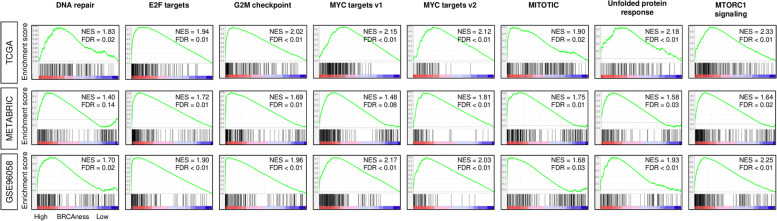
Hallmark GSEA of high BRCAness in breast cancer. Enrichment analysis of gene signaling pathways which have significant difference between low and high BRCAness consistently in three cohorts; TCGA, METABRIC, and GSE96058 cohorts. The top tertile was used as cutoff to divide high- or low- BRCAness groups. FDR, false discovery rate; NES, normalized enrichment score

As expected, high BRCAness significantly enriched DNA repair gene sets consistently in three cohorts (Fig. 3). Interestingly, BRCAness uniformly enriched five cell proliferation-related gene sets in the Hallmark collection; Mitotic spindle, MYC targets v1 and v2, G2M checkpoint, and E2F targets, as well as unfolded protein response, and MTORC1 signaling, consistently in three large cohorts.

### High BRCAness breast cancer was associated significantly with high cytolytic activity and with infiltration of anti-cancerous immune cells

**Fig. 4:**
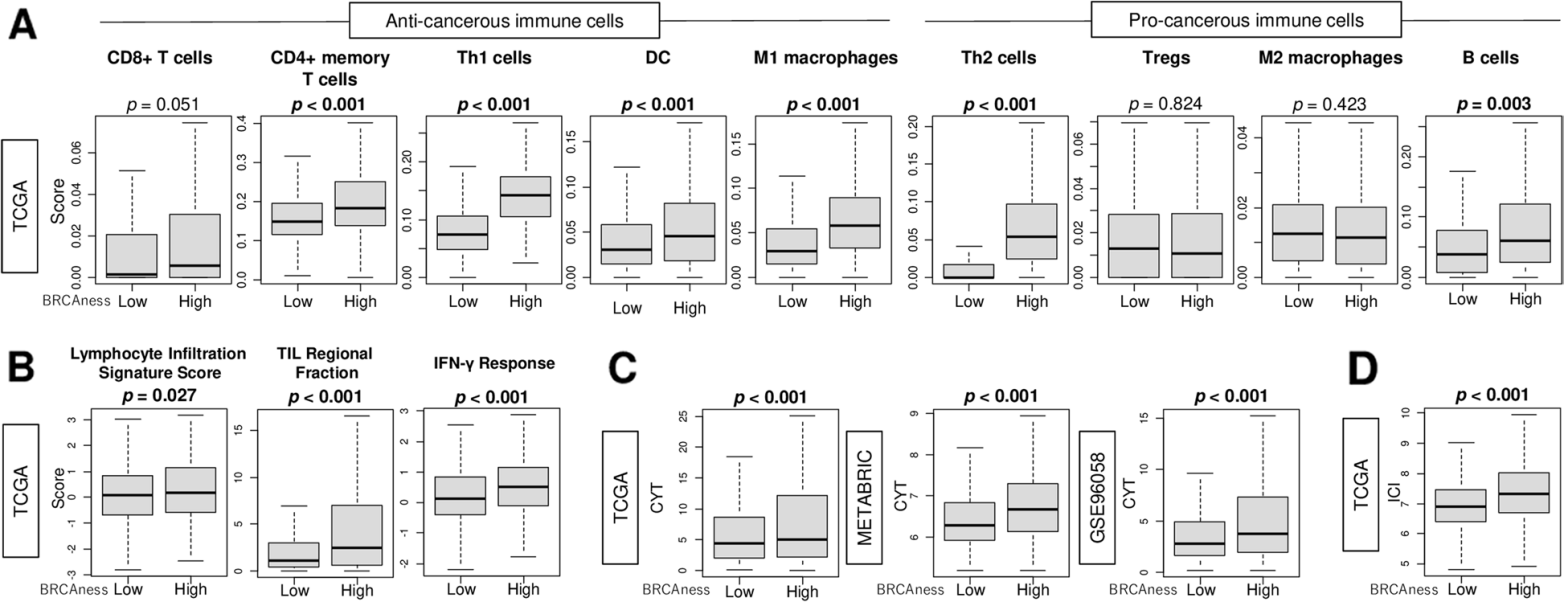
Breast cancer with a high BRCAness score was associated significantly with high levels of anti-cancerous immune cell infiltration, and cytolytic activity. Boxplots comparing low or high BRCAness breast cancers in the TCGA, METABRIC, and GSE96058 cohorts. **A** Infiltrating anti-cancerous immune cells; CD4^+^ memory T cells, CD8 T^+^ cells, Th1 cells, DC, and M1 macrophages, and pro-cancerous immune cells; Th2 cells, Tregs, M2 macrophages, and B cells, and **(B)** immune-cell-related score; lymphocyte infiltration signature, TIL regional fraction, and IFN-γ response, by low and high BRCAness tumors in the TCGA cohorts. **C** Cytolytic activity score (CYT) **(D)** Immune checkpoint index (ICI) score by low and high BRCAness score in the TCGA cohort. The top tertile was used as cutoff to divide high- or low- BRCAness groups. The Mann-Whitney U test was used to calculate *p*-values

Tumor-infiltrating immune cells are known to be attracted to the TME through neoantigens generated by tumor mutation burden [26]. We found that high BRCAness tumor was associated significantly with high infiltration of several anti-cancerous immune cells, such as M1 macrophages, T helper (Th) type 1 cells, CD4^+^ memory T cells, and dendritic cells (Fig. 4A; all *p* < 0.001), as well as B cells and Th2 cells (*p* = 0.002 and < 0.001, respectively), in TCGA (Fig. 4A). These results were validated by METABRIC and GSE96058 cohorts (Supplemental Fig. S1). M2 macrophage infiltration did not significantly differ between the two groups in any cohorts (Fig. 4A and Fig. S3). High BRCAness tumor was associated significantly with high infiltration of CD8^+^ T cells in METABRIC and GSE96058, although no significant association was observed in TCGA (Fig. 4A and Supplemental S1). High BRCAness tumor was significantly associated with several immune-related scores including lymphocyte infiltration signaling, tumor infiltrating lymphocytes regional fraction, and interferon (IFN)-γ response, in TCGA (Fig. 4B; *p* < 0.001, =0.027, and < 0.001, respectively). Further, high BRCAness tumor was associated significantly with high cytolytic activity (CYT) consistently in the three cohorts (Fig. 4C; all *p* < 0.001). Lastly, high BRCAness tumor was associated significantly with high level of immune checkpoint index (ICI) score, that represents the overall expression of immune checkpoint molecules in the TCGA cohort and validated in GSE96058 cohort (Fig. 4D and supplemental Fig. S4; both *p* < 0.001).

### Immune cells in the TME contributed to BRCAness in breast cancer

**Fig. 5:**
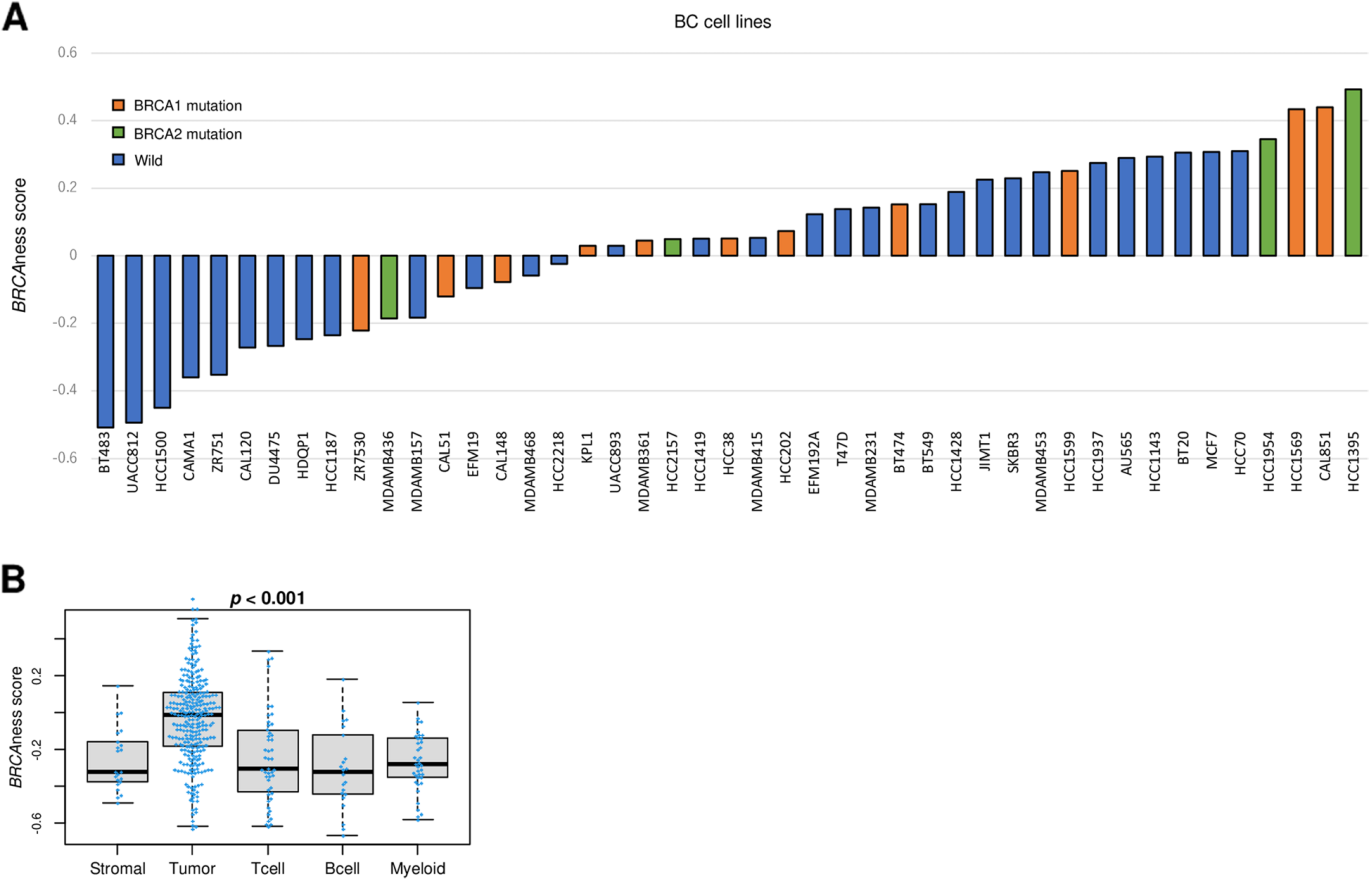
The association of the BRCAness score with BRCA mutation in breast cancer cell lines. **A** Bar plots of BRCAness score by 37 breast cancer cell lines in CCLE data base. Orange, green, and blue bars show BRCA1-mutation, BRCA2-mutation, and wildtype, respectively. **B** Boxplots of BRCAness score by different cells, including stromal cells, myeloid cells, B cells, T cells, and cancer cells, in bulk tumor in GSE75688 cohort. *P*-values were calculated by the Kruskal-Wallis test

We measured BRCAness in cell lines with or without BRCA mutations in Cancer Cell Line Encyclopedia database (CCLE; https://www.broadinstitute.org/ccle) and found that they did not match exactly (Fig. 5A). Supervised clustering analysis of the cell lines using BRCAness score gene expressions demonstrated similar result (Fig. S5). Given the difference in the association of BRCA mutation and BRCAness between human samples (Fig. 1) and cell lines (Fig. 5A), we hypothesized that the BRCAness is determined not only by cancer cells but also by the other cells in the TME. To this end, we utilized single cell sequence dataset from GSE75688 cohort and found that although BRCAness score was the highest in cancer cells, other cells in the TME including stromal, T cell, B cell, and myeloid cells, also contributed to the BRCAness score (Fig. 5B; *p* < 0.001).

### High BRCAness was associated significantly with triple-negative breast cancer (TNBC) and higher pathological grade, and good response to chemotherapy and PARP inhibitor in both ER-positive/HER2-negative breast cancer and TNBC

**Fig. 6:**
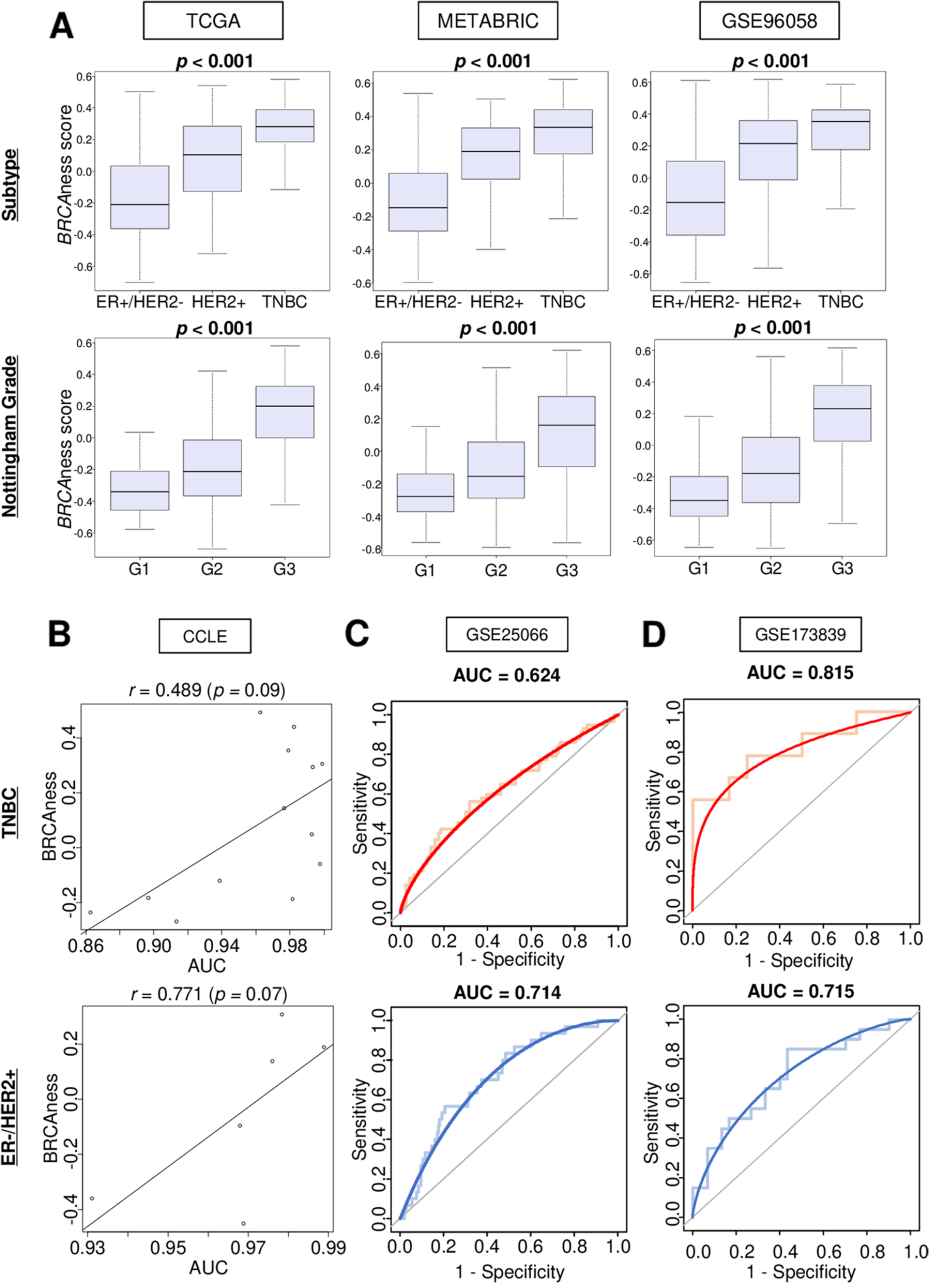
Association of BRCAness with clinical factors and drug response in breast cancer. **A** Box plots of BRCAness score by ER-positive/HER2-negative, HER2-positive, and TNBC, in METABRIC, TCGA, and GSE96058. The Kruskal-Wallis test was used to calculate *p*-values. **B** Correlation plots of the level of BRCAness and drug sensitivity AUC for olaparib in TNBC and ER-positive/HER2-negative breast cancer cell lines in CCLE data base. Spearman’s rank correlation test was used to perform the analysis. ROC of BRCAness score with AUC in TNBC and ER-positive/HER2-negative breast cancer in (**C**) GSE25066 cohort (Regimen; taxane and anthracycline) and (**D**) GSE173839 cohort (Regimen; Durvalumab and olaparib). AUC, area under the curve; ER, estrogen receptor; HER2, human epidermal growth factor receptor 2; ROC, Receiver operating characteristic; TNBC, triple negative breast cancer

BRCAness was significantly higher in TNBC compared to the other breast cancer subtypes consistently in three independent cohorts (Fig. 6A; *p* < 0.001). BRCAness was significantly associated with high Nottingham pathological grade consistently in 3 cohorts (Fig. 6A; all *p* < 0.001).

Next, we investigated whether BRCAness is associated with drug response. Correlation between BRCAness score and AUC of response to olaparib, a PARPi, was assessed using CCLE. BRCAness and AUC to olaparib showed positive correlation in both TNBC and ER-positive/HER2-negative subtypes (*r* = 0.489 and 0.771, respectively), although no significant difference was observed, most likely due to very small number of cell lines (Fig. 6A, both *p* > 0.05). In order to assess the clinical relevance, the association of BRCAness with response to neoadjuvant chemotherapy was also investigated. BRCAness score showed moderate correlation with pathological complete response (pCR) rate in both TNBC and ER-positive/HER2-negative breast cancer (Fig. 6B, AUC = 0.624 and 0.714, respectively) in GSE25066 cohort, which received anthracycline and taxane neoadjuvant chemotherapy (*n* = 508). Furthermore, it was strongly correlated with pCR after treatment with durvalumab and olaparib in both subtypes in GSE173839 cohort (Fig. 6C, AUC = 0.815 and 0.715, respectively). These results suggest that high BRCAness was associated with aggressive phenotype, and drug response particularly to PARPi olaparib.

## Discussion

The Phase 3 OlympiAD trial found significant improvement of overall response rate and progression-free survival in germline BRCA mutation metastatic breast cancer with Olaparib compared from chemotherapy [27]. Similarly, the phase 3 EMBRACA trial reported significant improvement in progression-free survival and response rate in germline BRCA mutation metastatic breast cancer with Talozaparib compared from chemotherapy [28]. OlympiA adjuvant phase 3 randomized trial found significant improvement in 3-year invasive and distant disease-free survival in germline BRCA mutation with oral Olaparib compared from placebo [29]. Olaparib Extended, phase II study showed that PARPi is effective for metastatic breast cancer with germline PALB2 or somatic BRCA mutations [30].

Reduction of BRCA expression by modulating E2F transcriptional factors, cyclin-dependent kinases changes in methylation of histones [1], or disruption of other DNA damage response effectors [31] can impair HRR resulting in BRCAness [32]; however, its phenotype remains poorly defined [3]. Somatic mutations of HRR genes were thought to associate with BRCAness [33]. Partial or total loss of these genes increases sensitivity to DNA cross-linkers and PARPis [34, 35]. These findings suggest that BRCAness may be determined by accumulation of many markers that each may be present in only a small fraction of tumors. Whole exome sequencing combined with transcriptome profiling has found that alterations in at least one HRR gene were present in about 50% of high grade serous ovarian cancer where this may be mediated by RNA polymerase regulator CDK12, which is required for the transcription of key HRR-related genes such as *BRCA1, ATR, FANCI, and FANCD2* [36]. Whole-exome and transcriptome profiling of 150 metastatic castration-resistant prostate cancer found that more than 19% of them have at least one mutation in *BRCA1, BRCA2, ATM and CDK12* [37]. To date, several studies utilized germline BRCA gene mutation-associated mutagenic gene signatures to identify BRCAness in BRCA wildtype tumors. Konstantinopoulos et al. generated a BRCAness gene expression profile using transcriptome and BRCA1 and/or BRCA2 mutations [38]. Larsen et al. generated a transcriptional signature to predict BRCA-mutated cancers [39]. Given the concept of BRCAness, it was reasonable to utilize a number of genes that impair HRR, rather than single genes such as *BRCA1* or *BRCA2*. We used mRNA expression of 34 genes to establish the BRCAness score. Among them, some genes have functions related to BRCAness shown in the study. For example, PSMD2 (26S proteasome non-ATPase regulatory subunit 2) is responsible for substrate recognition and binding. Disturbances in the signaling is involved in misfolded protein species and contribute to inflammatory response and systemic DNA damage responses leading to malignancies [40]. PTP (protein tyrosine phosphatase) family are known to be signaling molecules that regulate a variety of cellular processes including cell growth, differentiation, mitotic cycle, and oncogenic transformation, and propose to function as a tumor suppressor in cancer [41]. TRIP13 (Thyroid receptor-interacting protein 13) has a role in cell cycle arrest and cancer progression [42].

The novelty of our study is that we established a novel BRCAness score based on transcriptome validated with multiple independent large human cohorts. To establish the score, we first showed that the score reflects the biology of BRCAness in multiple ways. We were then able to show a clinically significant association of the score with response to PARPi using a completely different cohort. Our BRCAness score strongly correlated with biologically aggressive cancer that enriched all 5 cell proliferation-related gene sets and unfolded protein response signaling and associated with TNBC subtype. This is consistent with the previous reports that familial-BRCA1 mutant tumors segregate strongly with basal subtype [43, 44], which indicates that basal-type sporadic tumors and familial-BRCA1 tumors could have a similar biology [45]. Despite the fact that BRCA mutation is an important factor affecting DNA repair, there was no significant difference in any of the Hallmark gene set enrichment when comparing BRCA mutation with BRCA wildtype breast cancers. We speculated that this may be due to presence of cancers with BRCAness in the BRCA non-mutation group.

DNA damage and genomic instability are known to be closely related to immunity. BRCA mutated tumors have higher mitotic count and lymphocyte infiltration [46]. Unstable cancer genome creates various mutations that produce neoantigens [47]. Therefore, immunity may be particularly important in BRCA germline mutation carriers with dysfunctional DNA repair and HRD [48]. Melanoma and lung cancer with germline BRCA mutation are already known to be successful candidates for immune checkpoint inhibition due to their high tumor mutational burden [49]. Even though our BRCAness score was generated based on BRCA1 mutation tumors, this relationship was not always the case in breast cancer cell lines. This is because BRCAness is embodied not only by cancer cells, but also by immune and stromal cells that exist in the TME. Studies using cell lines are essential for unraveling the mechanisms; however, it is critical to recognize that it does not always mimic the human cancer with its TME. Our group has extensive experience in animal models including syngeneic models [50] and patient-derived xenograft mouse models using human breast cancer patient samples [51], however, none of them is able to completely replicate human tumors. On the other hand, in the analysis using human samples, the BRCAness score showed a strong correlation with drug response, especially, after treatment with PARPi in the TNBC subtype. Therefore, we cannot help but speculate that our BRCAness score that is generated using large cohorts with human tumors, is able to detect the group of patients who would benefit with the use of PARPi that cannot be identified just by testing for BRCA mutation, and therefore, hopefully can be used as a predictive biomarker not only for patient with BRCA mutation but also for patients who are BRCA wildtype in the future studies. This study is not free from limitations; this is a retrospective study using transcriptomics data alone and lacks protein confirmation using gold standards, such as flow cytometry or immunohistochemistry. Although there is no doubt that it will be ideal to obtain protein confirmation, we argue that even a gold standard such as immunohistochemistry is not almighty. Microscopic assessments by human judgment are prone to subjectivity and hence have limited reproducibility. It has been well demonstrated that variation in staining intensities [52] such as hotspots [53], and the mode of microscopic evaluation [54] are at least part of the reasons for the discordant results by the observers. Biomarkers that have been clinically utilized in recent years, such as Oncotype DX, use transcriptomic data, which enables objective evaluation of gene expression. Focusing on the transcriptome may provide hints to solve clinical problems, such as difficulty of grasping BRCAness from BRCA mutations alone. Another limitation what we found was the correlation between BRCAness and BRCA1 mutation of the tumor where we are unable to distinguish germline from somatic mutation. Because Olaparib is indicated for breast cancer patients with germline mutations, it is clinically important to make that distinction, although Olaparib has also shown clinical efficacy in tumors with somatic BRCA 1/2 mutation per the findings of the TBCRC 048 clinical trial [30]. We speculate that the BRCAness score may correlate with germline mutations since it strongly correlated with the response to Olaparib; however, this remains to be proven. Furthermore, a prospective clinical trial is essential to validate the use of BRCAness score as predictive biomarker for PARPi.

## Conclusions

We established novel BRCAness score using mRNA expression of BRCA-mutation-related genes, and found that it associates with DNA repair, immunity, mutation load, and drug response in breast cancer.

## Supplementary Information

**Additional file 1: Table S1.** Gene included in the Molecular Signatures Database (MSigDB) Hallmark DNA repair gene set. **Table S2.** Thirty-four genes significantly associated with BRCA1-mutation consistently in both TCGA and METABRIC cohorts (AUC > 0.65). **Figure S1.** Histogram of BRCAness score in the TCGA and METABRIC cohorts. **Figure S2.** Association of BRCAness score with BRCA2 mutation in breast cancer. **Figure S3.** Breast cancer with a high BRCAness score was significantly associated with high levels of anti-cancerous immune cells infiltration. **Figure S4.** Breast cancer with a high BRCAness score was significantly associated with high levels of immune checkpoint index (ICI) score in the GSE96058 cohort. **Figure S5.** The association of mRNA expression of the BRCAness gene set with BRCA mutation in breast cancer cell lines.

## Data Availability

All the cohorts/datasets used in this study; Molecular Taxonomy of Breast Cancer International Consortium (METABRIC), GSE96058, The Cancer Genome Atlas (TCGA), The Cancer Cell Line Encyclopedia (CCLE), GSE25066, and GSE173839, are all publicly available without any restrictions via cBioportal or Gene Expression Omnibus (GEO).
