# Supplementary material for: Development of a novel BRCAness score that predicts response to PARP inhibitors
Source: Biomark Res. 2022 Nov 12;10:80. doi: 10.1186/s40364-022-00427-8 (PMC9652967; doi:10.1186/s40364-022-00427-8)
Supplement: Supplementary file 1 — Additional file 1: Table S1. Gene included in the Molecular Signatures Database (MSigDB) Hallmark DNA repair gene set. Table S2. Thirty-four genes significantly associated with BRCA1-mutation consistently in both TCGA and METABRIC cohorts (AUC > 0.65). Figure S1. Histogram of BRCAness score in the TCGA and METABRIC cohorts. Figure S2. Association of BRCAness score with BRCA2 mutation in breast cancer. Figure S3. Breast cancer with a high BRCAness score was significantly associated with high levels of anti-cancerous immune cells infiltration. Figure S4. Breast cancer with a high BRCAness score was significantly associated with high levels of immune checkpoint index (ICI) score in the GSE96058 cohort. Figure S5. The association of mRNA expression of the BRCAness gene set with BRCA mutation in breast cancer cell lines. [file 40364_2022_427_MOESM1_ESM.docx]

**SUPPLEMENTAL MATERIALS**

Oshi et al. “Development of a novel BRCAness score that predicts response to PARP inhibitors”

Contents:

**Table S1:** Gene included in the Molecular Signatures Database (MSigDB) Hallmark DNA repair gene set.

**Table S2:** Thirty-four genes significantly associated with BRCA1-mutation consistently in both TCGA and METABRIC cohorts (AUC > 0.65).

**Figure S1:** Histogram of BRCAness score in the TCGA and METABRIC cohorts.

**Figure S2:** Association of BRCAness score with BRCA2 mutation in breast cancer.

**Figure S3:** Breast cancer with a high BRCAness score was significantly associated with high levels of anti-cancerous immune cells infiltration.

**Figure S4:** Breast cancer with a high BRCAness score was significantly associated with high levels of immune checkpoint index (ICI) score in the GSE96058 cohort.

**Figure S5:** The association of mRNA expression of the BRCAness gene set with BRCA mutation in breast cancer cell lines.

**Table S1: Gene included in the Molecular Signatures Database (MSigDB) Hallmark DNA repair gene set.**

| *Gene Symbol* | *Gene description* |
| --- | --- |
| [AAAS](http://ensembl.org/Homo_sapiens/Gene/Summary?db=core;g=AAAS) | aladin WD repeat nucleoporin |
| [ADA](http://ensembl.org/Homo_sapiens/Gene/Summary?db=core;g=ADA) | adenosine deaminase |
| [ADCY6](http://ensembl.org/Homo_sapiens/Gene/Summary?db=core;g=ADCY6) | adenylate cyclase 6 |
| [ADRM1](http://ensembl.org/Homo_sapiens/Gene/Summary?db=core;g=ADRM1) | adhesion regulating molecule 1 |
| [AK1](http://ensembl.org/Homo_sapiens/Gene/Summary?db=core;g=AK1) | adenylate kinase 1 |
| [AK3](http://ensembl.org/Homo_sapiens/Gene/Summary?db=core;g=AK3) | adenylate kinase 3 |
| [APRT](http://ensembl.org/Homo_sapiens/Gene/Summary?db=core;g=APRT) | adenine phosphoribosyltransferase |
| [ARL6IP1](http://ensembl.org/Homo_sapiens/Gene/Summary?db=core;g=ARL6IP1) | ADP ribosylation factor like GTPase 6 interacting protein 1 |
| [BCAM](http://ensembl.org/Homo_sapiens/Gene/Summary?db=core;g=BCAM) | basal cell adhesion molecule |
| [BCAP31](http://ensembl.org/Homo_sapiens/Gene/Summary?db=core;g=BCAP31) | B cell receptor associated protein 31 |
| [BOLA2](http://ensembl.org/Homo_sapiens/Gene/Summary?db=core;g=BOLA2) | bolA family member 2 |
| [BRF2](http://ensembl.org/Homo_sapiens/Gene/Summary?db=core;g=BRF2) | BRF2 RNA polymerase III transcription initiation factor subunit |
| [MPC2](http://ensembl.org/Homo_sapiens/Gene/Summary?db=core;g=MPC2) | mitochondrial pyruvate carrier 2 |
| [CANT1](http://ensembl.org/Homo_sapiens/Gene/Summary?db=core;g=CANT1) | calcium activated nucleotidase 1 |
| [CCNO](http://ensembl.org/Homo_sapiens/Gene/Summary?db=core;g=CCNO) | cyclin O |
| [CDA](http://ensembl.org/Homo_sapiens/Gene/Summary?db=core;g=CDA) | cytidine deaminase |
| [CETN2](http://ensembl.org/Homo_sapiens/Gene/Summary?db=core;g=CETN2) | centrin 2 |
| [CLP1](http://ensembl.org/Homo_sapiens/Gene/Summary?db=core;g=CLP1) | cleavage factor polyribonucleotide kinase subunit 1 |
| [CMPK2](http://ensembl.org/Homo_sapiens/Gene/Summary?db=core;g=CMPK2) | cytidine/uridine monophosphate kinase 2 |
| [NELFB](http://ensembl.org/Homo_sapiens/Gene/Summary?db=core;g=NELFB) | negative elongation factor complex member B |
| [COX17](http://ensembl.org/Homo_sapiens/Gene/Summary?db=core;g=COX17) | cytochrome c oxidase copper chaperone COX17 |
| [CSTF3](http://ensembl.org/Homo_sapiens/Gene/Summary?db=core;g=CSTF3) | cleavage stimulation factor subunit 3 |
| [DAD1](http://ensembl.org/Homo_sapiens/Gene/Summary?db=core;g=DAD1) | defender against cell death 1 |
| [DCTN4](http://ensembl.org/Homo_sapiens/Gene/Summary?db=core;g=DCTN4) | dynactin subunit 4 |
| [DDB1](http://ensembl.org/Homo_sapiens/Gene/Summary?db=core;g=DDB1) | damage specific DNA binding protein 1 |
| [DDB2](http://ensembl.org/Homo_sapiens/Gene/Summary?db=core;g=DDB2) | damage specific DNA binding protein 2 |
| [GSDME](http://ensembl.org/Homo_sapiens/Gene/Summary?db=core;g=GSDME) | gasdermin E |
| [DGCR8](http://ensembl.org/Homo_sapiens/Gene/Summary?db=core;g=DGCR8) | DGCR8 microprocessor complex subunit |
| [DGUOK](http://ensembl.org/Homo_sapiens/Gene/Summary?db=core;g=DGUOK) | deoxyguanosine kinase |
| [DUT](http://ensembl.org/Homo_sapiens/Gene/Summary?db=core;g=DUT) | deoxyuridine triphosphatase |
| [EDF1](http://ensembl.org/Homo_sapiens/Gene/Summary?db=core;g=EDF1) | endothelial differentiation related factor 1 |
| [EIF1B](http://ensembl.org/Homo_sapiens/Gene/Summary?db=core;g=EIF1B) | eukaryotic translation initiation factor 1B |
| [AGO4](http://ensembl.org/Homo_sapiens/Gene/Summary?db=core;g=AGO4) | argonaute RISC component 4 |
| [ELL](http://ensembl.org/Homo_sapiens/Gene/Summary?db=core;g=ELL) | elongation factor for RNA polymerase II |
| [ERCC1](http://ensembl.org/Homo_sapiens/Gene/Summary?db=core;g=ERCC1) | ERCC excision repair 1, endonuclease non-catalytic subunit |
| [ERCC2](http://ensembl.org/Homo_sapiens/Gene/Summary?db=core;g=ERCC2) | ERCC excision repair 2, TFIIH core complex helicase subunit |
| [ERCC3](http://ensembl.org/Homo_sapiens/Gene/Summary?db=core;g=ERCC3) | ERCC excision repair 3, TFIIH core complex helicase subunit |
| [ERCC4](http://ensembl.org/Homo_sapiens/Gene/Summary?db=core;g=ERCC4) | ERCC excision repair 4, endonuclease catalytic subunit |
| [ERCC5](http://ensembl.org/Homo_sapiens/Gene/Summary?db=core;g=ERCC5) | ERCC excision repair 5, endonuclease |
| [ERCC8](http://ensembl.org/Homo_sapiens/Gene/Summary?db=core;g=ERCC8) | ERCC excision repair 8, CSA ubiquitin ligase complex subunit |
| [FEN1](http://ensembl.org/Homo_sapiens/Gene/Summary?db=core;g=FEN1) | flap structure-specific endonuclease 1 |
| [GMPR2](http://ensembl.org/Homo_sapiens/Gene/Summary?db=core;g=GMPR2) | guanosine monophosphate reductase 2 |
| [GPX4](http://ensembl.org/Homo_sapiens/Gene/Summary?db=core;g=GPX4) | glutathione peroxidase 4 |
| [GTF2A2](http://ensembl.org/Homo_sapiens/Gene/Summary?db=core;g=GTF2A2) | general transcription factor IIF subunit 2 |
| [GTF2B](http://ensembl.org/Homo_sapiens/Gene/Summary?db=core;g=GTF2B) | general transcription factor IIB |
| [GTF2F1](http://ensembl.org/Homo_sapiens/Gene/Summary?db=core;g=GTF2F1) | general transcription factor IIF subunit 1 |
| [GTF2H1](http://ensembl.org/Homo_sapiens/Gene/Summary?db=core;g=GTF2H1) | general transcription factor IIH subunit 1 |
| [GTF2H3](http://ensembl.org/Homo_sapiens/Gene/Summary?db=core;g=GTF2H3) | general transcription factor IIH subunit 3 |
| [GTF2H5](http://ensembl.org/Homo_sapiens/Gene/Summary?db=core;g=GTF2H5) | general transcription factor IIH subunit 5 |
| [GTF3C5](http://ensembl.org/Homo_sapiens/Gene/Summary?db=core;g=GTF3C5) | general transcription factor IIIC subunit 5 |
| [GUK1](http://ensembl.org/Homo_sapiens/Gene/Summary?db=core;g=GUK1) | guanylate kinase 1 |
| [HCLS1](http://ensembl.org/Homo_sapiens/Gene/Summary?db=core;g=HCLS1) | hematopoietic cell-specific Lyn substrate 1 |
| [HPRT1](http://ensembl.org/Homo_sapiens/Gene/Summary?db=core;g=HPRT1) | hypoxanthine phosphoribosyltransferase 1 |
| [IMPDH2](http://ensembl.org/Homo_sapiens/Gene/Summary?db=core;g=IMPDH2) | inosine monophosphate dehydrogenase 2 |
| [ITPA](http://ensembl.org/Homo_sapiens/Gene/Summary?db=core;g=ITPA) | inosine triphosphatase |
| [LIG1](http://ensembl.org/Homo_sapiens/Gene/Summary?db=core;g=LIG1) | DNA ligase 1 |
| [MPG](http://ensembl.org/Homo_sapiens/Gene/Summary?db=core;g=MPG) | N-methylpurine DNA glycosylase |
| [MRPL40](http://ensembl.org/Homo_sapiens/Gene/Summary?db=core;g=MRPL40) | mitochondrial ribosomal protein L40 |
| [NCBP2](http://ensembl.org/Homo_sapiens/Gene/Summary?db=core;g=NCBP2) | nuclear cap binding protein subunit 2 |
| [NFX1](http://ensembl.org/Homo_sapiens/Gene/Summary?db=core;g=NFX1) | nuclear transcription factor, X-box binding 1 |
| [NME1](http://ensembl.org/Homo_sapiens/Gene/Summary?db=core;g=NME1) | NME/NM23 nucleoside diphosphate kinase 1 |
| [NME3](http://ensembl.org/Homo_sapiens/Gene/Summary?db=core;g=NME3) | NME/NM23 nucleoside diphosphate kinase 3 |
| [NME4](http://ensembl.org/Homo_sapiens/Gene/Summary?db=core;g=NME4) | NME/NM23 nucleoside diphosphate kinase 4 |
| [NPR2](http://ensembl.org/Homo_sapiens/Gene/Summary?db=core;g=NPR2) | natriuretic peptide receptor 2 |
| [NT5C](http://ensembl.org/Homo_sapiens/Gene/Summary?db=core;g=NT5C) | 5', 3'-nucleotidase, cytosolic |
| [NT5C3A](http://ensembl.org/Homo_sapiens/Gene/Summary?db=core;g=NT5C3A) | 5'-nucleotidase, cytosolic IIIA |
| [NUDT21](http://ensembl.org/Homo_sapiens/Gene/Summary?db=core;g=NUDT21) | nudix hydrolase 21 |
| [NUDT9](http://ensembl.org/Homo_sapiens/Gene/Summary?db=core;g=NUDT9) | nudix hydrolase 9 |
| [PCNA](http://ensembl.org/Homo_sapiens/Gene/Summary?db=core;g=PCNA) | proliferating cell nuclear antigen |
| [PDE4B](http://ensembl.org/Homo_sapiens/Gene/Summary?db=core;g=PDE4B) | phosphodiesterase 4B |
| [PDE6G](http://ensembl.org/Homo_sapiens/Gene/Summary?db=core;g=PDE6G) | phosphodiesterase 6G |
| [PNP](http://ensembl.org/Homo_sapiens/Gene/Summary?db=core;g=PNP) | purine nucleoside phosphorylase |
| [POLA1](http://ensembl.org/Homo_sapiens/Gene/Summary?db=core;g=POLA1) | DNA polymerase alpha 1, catalytic subunit |
| [POLA2](http://ensembl.org/Homo_sapiens/Gene/Summary?db=core;g=POLA2) | DNA polymerase alpha 2, accessory subunit |
| [POLB](http://ensembl.org/Homo_sapiens/Gene/Summary?db=core;g=POLB) | DNA polymerase beta |
| [POLD1](http://ensembl.org/Homo_sapiens/Gene/Summary?db=core;g=POLD1) | DNA polymerase delta 1, catalytic subunit |
| [POLD3](http://ensembl.org/Homo_sapiens/Gene/Summary?db=core;g=POLD3) | DNA polymerase delta 3, accessory subunit |
| [POLD4](http://ensembl.org/Homo_sapiens/Gene/Summary?db=core;g=POLD4) | DNA polymerase delta 4, accessory subunit |
| [POLE4](http://ensembl.org/Homo_sapiens/Gene/Summary?db=core;g=POLE4) | DNA polymerase epsilon 4, accessory subunit |
| [POLH](http://ensembl.org/Homo_sapiens/Gene/Summary?db=core;g=POLH) | DNA polymerase eta |
| [POLL](http://ensembl.org/Homo_sapiens/Gene/Summary?db=core;g=POLL) | DNA polymerase lambda |
| [POLR1C](http://ensembl.org/Homo_sapiens/Gene/Summary?db=core;g=POLR1C) | RNA polymerase I and III subunit C |
| [POLR1D](http://ensembl.org/Homo_sapiens/Gene/Summary?db=core;g=POLR1D) | RNA polymerase I and III subunit D |
| [POLR2A](http://ensembl.org/Homo_sapiens/Gene/Summary?db=core;g=POLR2A) | RNA polymerase II subunit A |
| [POLR2C](http://ensembl.org/Homo_sapiens/Gene/Summary?db=core;g=POLR2C) | RNA polymerase II subunit C |
| [POLR2D](http://ensembl.org/Homo_sapiens/Gene/Summary?db=core;g=POLR2D) | RNA polymerase II subunit D |
| [POLR2E](http://ensembl.org/Homo_sapiens/Gene/Summary?db=core;g=POLR2E) | RNA polymerase II subunit E |
| [POLR2F](http://ensembl.org/Homo_sapiens/Gene/Summary?db=core;g=POLR2F) | RNA polymerase II subunit F |
| [POLR2G](http://ensembl.org/Homo_sapiens/Gene/Summary?db=core;g=POLR2G) | RNA polymerase II subunit G |
| [POLR2H](http://ensembl.org/Homo_sapiens/Gene/Summary?db=core;g=POLR2H) | RNA polymerase II subunit H |
| [POLR2I](http://ensembl.org/Homo_sapiens/Gene/Summary?db=core;g=POLR2I) | RNA polymerase II subunit I |
| [POLR2J](http://ensembl.org/Homo_sapiens/Gene/Summary?db=core;g=POLR2J) | RNA polymerase II subunit J |
| [POLR2K](http://ensembl.org/Homo_sapiens/Gene/Summary?db=core;g=POLR2K) | RNA polymerase II subunit K |
| [POLR3C](http://ensembl.org/Homo_sapiens/Gene/Summary?db=core;g=POLR3C) | RNA polymerase III subunit C |
| [POLR3GL](http://ensembl.org/Homo_sapiens/Gene/Summary?db=core;g=POLR3GL) | RNA polymerase III subunit G like |
| [POM121](http://ensembl.org/Homo_sapiens/Gene/Summary?db=core;g=POM121) | POM121 transmembrane nucleoporin |
| [PRIM1](http://ensembl.org/Homo_sapiens/Gene/Summary?db=core;g=PRIM1) | DNA primase subunit 1 |
| [RAD51](http://ensembl.org/Homo_sapiens/Gene/Summary?db=core;g=RAD51) | RAD51 recombinase |
| [RAD52](http://ensembl.org/Homo_sapiens/Gene/Summary?db=core;g=RAD52) | RAD52 homolog, DNA repair protein |
| [RAE1](http://ensembl.org/Homo_sapiens/Gene/Summary?db=core;g=RAE1) | ribonucleic acid export 1 |
| [RALA](http://ensembl.org/Homo_sapiens/Gene/Summary?db=core;g=RALA) | RAS like proto-oncogene A |
| [RBX1](http://ensembl.org/Homo_sapiens/Gene/Summary?db=core;g=RBX1) | ring-box 1 |
| [NELFE](http://ensembl.org/Homo_sapiens/Gene/Summary?db=core;g=NELFE) | negative elongation factor complex member E |
| [REV3L](http://ensembl.org/Homo_sapiens/Gene/Summary?db=core;g=REV3L) | REV3 like, DNA directed polymerase zeta catalytic subunit |
| [RFC2](http://ensembl.org/Homo_sapiens/Gene/Summary?db=core;g=RFC2) | replication factor C subunit 2 |
| [RFC3](http://ensembl.org/Homo_sapiens/Gene/Summary?db=core;g=RFC3) | replication factor C subunit 3 |
| [RFC4](http://ensembl.org/Homo_sapiens/Gene/Summary?db=core;g=RFC4) | replication factor C subunit 4 |
| [RFC5](http://ensembl.org/Homo_sapiens/Gene/Summary?db=core;g=RFC5) | replication factor C subunit 5 |
| [RNMT](http://ensembl.org/Homo_sapiens/Gene/Summary?db=core;g=RNMT) | RNA guanine-7 methyltransferase |
| [RPA2](http://ensembl.org/Homo_sapiens/Gene/Summary?db=core;g=RPA2) | replication protein A2 |
| [RPA3](http://ensembl.org/Homo_sapiens/Gene/Summary?db=core;g=RPA3) | replication protein A3 |
| [RRM2B](http://ensembl.org/Homo_sapiens/Gene/Summary?db=core;g=RRM2B) | ribonucleotide reductase regulatory TP53 inducible subunit M2B |
| [SAC3D1](http://ensembl.org/Homo_sapiens/Gene/Summary?db=core;g=SAC3D1) | SAC3 domain containing 1 |
| [SDCBP](http://ensembl.org/Homo_sapiens/Gene/Summary?db=core;g=SDCBP) | syndecan binding protein |
| [SEC61A1](http://ensembl.org/Homo_sapiens/Gene/Summary?db=core;g=SEC61A1) | SEC61 translocon subunit alpha 1 |
| [SF3A3](http://ensembl.org/Homo_sapiens/Gene/Summary?db=core;g=SF3A3) | splicing factor 3a subunit 3 |
| [SMAD5](http://ensembl.org/Homo_sapiens/Gene/Summary?db=core;g=SMAD5) | SMAD family member 5 |
| [SNAPC4](http://ensembl.org/Homo_sapiens/Gene/Summary?db=core;g=SNAPC4) | small nuclear RNA activating complex polypeptide 4 |
| [SNAPC5](http://ensembl.org/Homo_sapiens/Gene/Summary?db=core;g=SNAPC5) | small nuclear RNA activating complex polypeptide 5 |
| [SRSF6](http://ensembl.org/Homo_sapiens/Gene/Summary?db=core;g=SRSF6) | small nuclear RNA activating complex polypeptide 6 |
| [SSRP1](http://ensembl.org/Homo_sapiens/Gene/Summary?db=core;g=SSRP1) | structure specific recognition protein 1 |
| [STX3](http://ensembl.org/Homo_sapiens/Gene/Summary?db=core;g=STX3) | syntaxin 3 |
| [SUPT4H1](http://ensembl.org/Homo_sapiens/Gene/Summary?db=core;g=SUPT4H1) | SPT4 homolog, DSIF elongation factor subunit |
| [SUPT5H](http://ensembl.org/Homo_sapiens/Gene/Summary?db=core;g=SUPT5H) | SPT5 homolog, DSIF elongation factor subunit |
| [SURF1](http://ensembl.org/Homo_sapiens/Gene/Summary?db=core;g=SURF1) | SURF1 cytochrome c oxidase assembly factor |
| [TAF10](http://ensembl.org/Homo_sapiens/Gene/Summary?db=core;g=TAF10) | TATA-box binding protein associated factor 10 |
| [TAF12](http://ensembl.org/Homo_sapiens/Gene/Summary?db=core;g=TAF12) | TATA-box binding protein associated factor 12 |
| [TAF13](http://ensembl.org/Homo_sapiens/Gene/Summary?db=core;g=TAF13) | TATA-box binding protein associated factor 13 |
| [TAF1C](http://ensembl.org/Homo_sapiens/Gene/Summary?db=core;g=TAF1C) | TATA-box binding protein associated factor, RNA polymerase I subunit C |
| [TAF6](http://ensembl.org/Homo_sapiens/Gene/Summary?db=core;g=TAF6) | TATA-box binding protein associated factor 6 |
| [TAF9](http://ensembl.org/Homo_sapiens/Gene/Summary?db=core;g=TAF9) | TATA-box binding protein associated factor 9 |
| [TARBP2](http://ensembl.org/Homo_sapiens/Gene/Summary?db=core;g=TARBP2) | TARBP2 subunit of RISC loading complex |
| [ELOA](http://ensembl.org/Homo_sapiens/Gene/Summary?db=core;g=ELOA) | elongin A |
| [NELFCD](http://ensembl.org/Homo_sapiens/Gene/Summary?db=core;g=NELFCD) | negative elongation factor complex member C/D |
| [ALYREF](http://ensembl.org/Homo_sapiens/Gene/Summary?db=core;g=ALYREF) | Aly/REF export factor |
| [TK2](http://ensembl.org/Homo_sapiens/Gene/Summary?db=core;g=TK2) | thymidine kinase 2 |
| [TMED2](http://ensembl.org/Homo_sapiens/Gene/Summary?db=core;g=TMED2) | transmembrane p24 trafficking protein 2 |
| [TP53](http://ensembl.org/Homo_sapiens/Gene/Summary?db=core;g=TP53) | tumor protein p53 |
| [TSG101](http://ensembl.org/Homo_sapiens/Gene/Summary?db=core;g=TSG101) | tumor susceptibility 101 |
| [TYMS](http://ensembl.org/Homo_sapiens/Gene/Summary?db=core;g=TYMS) | thymidylate synthetase |
| [UMPS](http://ensembl.org/Homo_sapiens/Gene/Summary?db=core;g=UMPS) | uridine monophosphate synthetase |
| [UPF3B](http://ensembl.org/Homo_sapiens/Gene/Summary?db=core;g=UPF3B) | UPF3B regulator of nonsense mediated mRNA decay |
| [USP11](http://ensembl.org/Homo_sapiens/Gene/Summary?db=core;g=USP11) | ubiquitin specific peptidase 11 |
| [VPS28](http://ensembl.org/Homo_sapiens/Gene/Summary?db=core;g=VPS28) | VPS28 subunit of ESCRT-I |
| [VPS37B](http://ensembl.org/Homo_sapiens/Gene/Summary?db=core;g=VPS37B) | VPS37B subunit of ESCRT-I |
| [VPS37D](http://ensembl.org/Homo_sapiens/Gene/Summary?db=core;g=VPS37D) | VPS37D subunit of ESCRT-I |
| [XPC](http://ensembl.org/Homo_sapiens/Gene/Summary?db=core;g=XPC) | XPC complex subunit, DNA damage recognition and repair factor |
| [ZNF707](http://ensembl.org/Homo_sapiens/Gene/Summary?db=core;g=ZNF707) | zinc finger protein 707 |
| [ZNRD1](http://ensembl.org/Homo_sapiens/Gene/Summary?db=core;g=ZNRD1) | zinc ribbon domain containing 1 |
| [ZWINT](http://ensembl.org/Homo_sapiens/Gene/Summary?db=core;g=ZWINT) | ZW10 interacting kinetochore protein |

**Table S2: Thirty-four genes significantly associated with BRCA1-mutation consistently in both TCGA and METABRIC cohorts (AUC > 0.65)**

|  | AUC | |
| --- | --- | --- |
| Gene | TCGA | METABRIC |
| **ADSL** | 0.66796 | 0.651774 |
| **AP2M1** | 0.656089 | 0.796225 |
| **ATP1A4** | 0.673562 | 0.661939 |
| **CBX2** | 0.652369 | 0.651347 |
| **CCDC125** | 0.665253 | 0.681951 |
| **CDC45** | 0.65745 | 0.657425 |
| **CDCA2** | 0.664761 | 0.674202 |
| **CENPO** | 0.68413 | 0.661015 |
| **CREB3L2** | 0.675929 | 0.656537 |
| **DNAJB11** | 0.667301 | 0.687105 |
| **ECE2** | 0.696007 | 0.698959 |
| **FAM136A** | 0.681191 | 0.663361 |
| **GORASP2** | 0.664543 | 0.661406 |
| **JOSD1** | 0.669234 | 0.710955 |
| **KCTD3** | 0.659752 | 0.693183 |
| **KRT222** | 0.655706 | 0.683479 |
| **LRRC28** | 0.692606 | 0.661708 |
| **MAOA** | 0.654381 | 0.701749 |
| **MAPT** | 0.658594 | 0.668302 |
| **MCM5** | 0.685469 | 0.681915 |
| **OTX1** | 0.676146 | 0.65739 |
| **POLG** | 0.657841 | 0.650459 |
| **POLR2H** | 0.659737 | 0.651169 |
| **PPP1R14B** | 0.654294 | 0.654191 |
| **PRC1** | 0.673432 | 0.659096 |
| **PRSS53** | 0.658492 | 0.688811 |
| **PSAT1** | 0.651182 | 0.660873 |
| **PSMD2** | 0.685722 | 0.720587 |
| **PTPRT** | 0.66028 | 0.672034 |
| **SNRPA1** | 0.658927 | 0.659025 |
| **ST3GAL4** | 0.685939 | 0.727447 |
| **TMEM150B** | 0.685997 | 0.698159 |
| **TRIP13** | 0.660143 | 0.658847 |
| **WARS** | 0.650979 | 0.681986 |

*AUC, area under the curve.

**
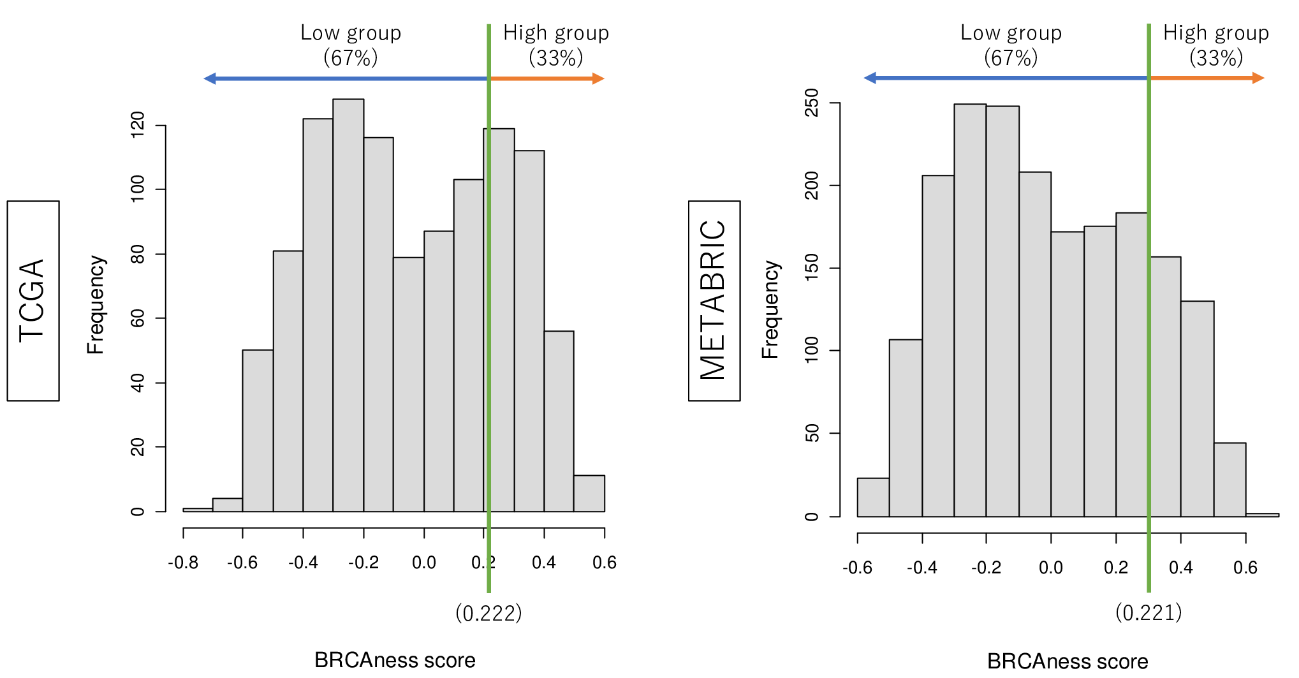
**

**Figure S1: Histogram of BRCAness score in the TCGA and METABRIC cohorts.**

**
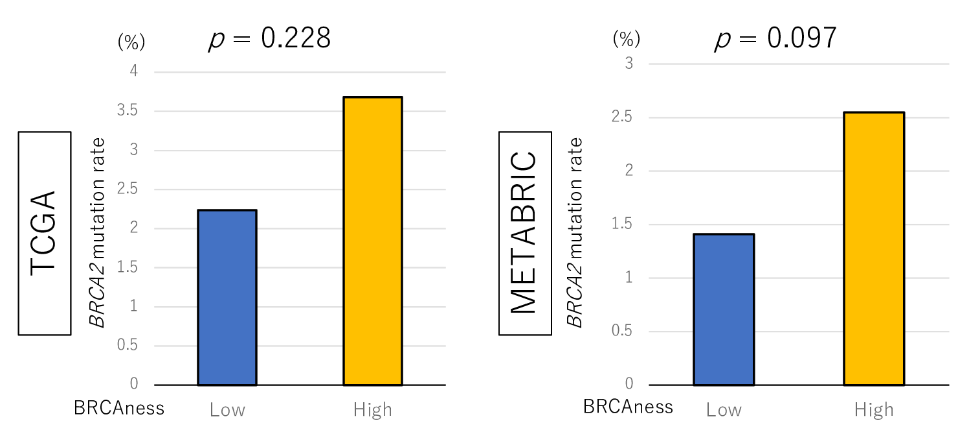
**

**Figure S2: Association of BRCAness score with BRCA2 mutation in breast cancer.** Bar plots of BRCA2 mutation rates by low or high BRCAness in TCGA and METABRIC cohorts. Fisher’s exact test was used to calculate the *p*-values. The top tertile was used as a cutoff to divide into high- or low- BRCAness groups.


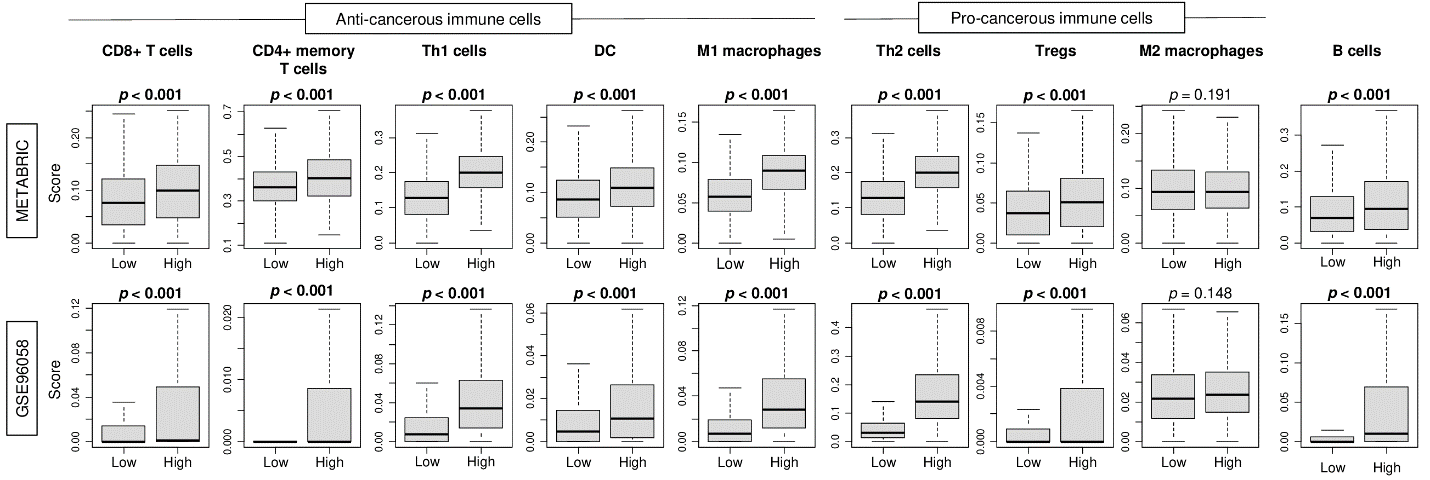


**Figure S3:** **Breast cancer with a high BRCAness score was significantly associated with high levels of anti-cancerous immune cells infiltration.** Boxplots of infiltrating fraction of anti-cancerous immune cells; CD8 T+ cells, CD4+ memory T cells, Th1 cells, DC, and M1 macrophages, and pro-cancerous immune cells; Th2 cells, Tregs, and M2 macrophages, and B cells in the METABRIC and GSE96058 cohorts. The top one third was used as a cut-off to divide two score groups. *p*-values were calculated by the Mann-Whitney U test.


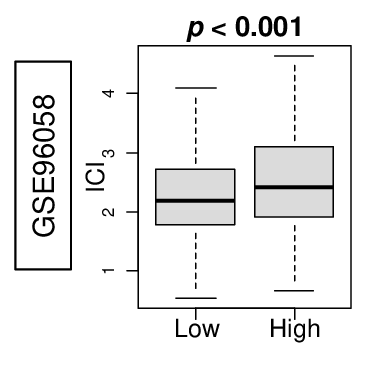


**Figure S4: Breast cancer with a high BRCAness score was significantly associated with high levels of immune checkpoint index (ICI) score in the GSE96058 cohort.** Boxplots of ICI score by low and high BRCAness score. The top one third was used as a cut-off to divide two score groups. *p*-values were calculated by the Mann-Whitney U test.


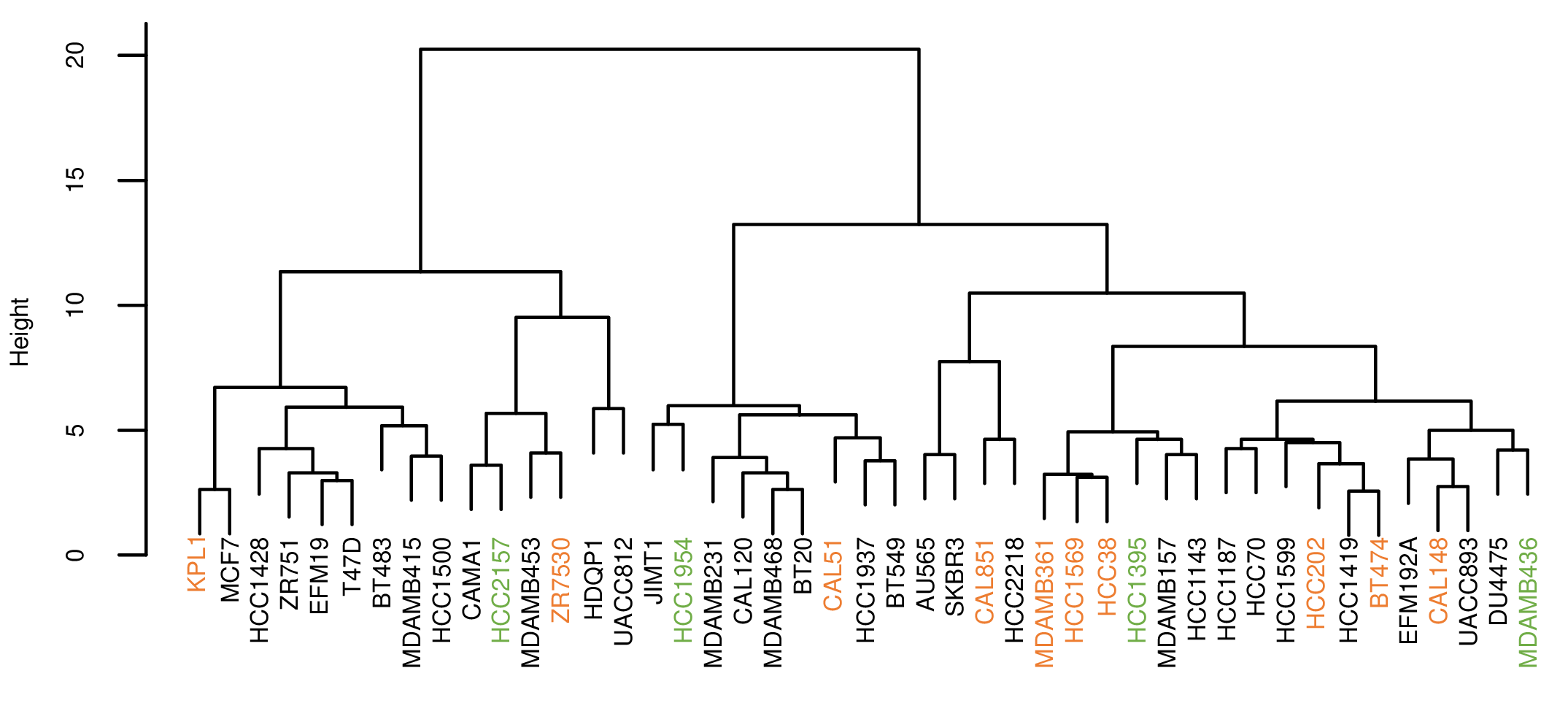


**Figure S5: The association of mRNA expression of the BRCAness gene set with BRCA mutation in breast cancer cell lines.** Cluster dendrogram of the cell lines using mRNA expression of BRCAness gene sets in CCLE data base. Orange and green show BRCA1-mutation, and BRCA2-mutation, respectively.
